# Synthesized flavone attenuates diabetes‐induced neurodegeneration through regulation of oxidative stress and metabolic‐neurodegenerative molecular pathways

**DOI:** 10.1002/ibra.70019

**Published:** 2026-04-18

**Authors:** Karishma Sen, Anita Sakarwal, Heera Ram, Suman K. Saha, Nirmal K. Rana, Dharmveer Yadav, Sunil Dutt Shukla, Mukesh Kumar Yadav, Balachandar Vellingiri, Vikas Kumar

**Affiliations:** ^1^ Department of Zoology Jai Narain Vyas University Jodhpur Rajasthan India; ^2^ Department of Chemistry Indian Institute of Technology Jodhpur Rajasthan India; ^3^ Department of Biochemistry All India Institute of Medical Sciences Jodhpur Rajasthan India; ^4^ Department of Zoology, Meera Girls College MLS University Udaipur Rajasthan India; ^5^ Department of Microbiology Central University of Punjab Bhatinda Punjab India; ^6^ Department of Zoology Central University of Punjab Bhatinda Punjab India; ^7^ Department of Pharmaceutical Sciences, SHUATS Natural Product Drug Discovery Laboratory Prayagraj India; ^8^ University Centre for Research and Development, Chandigarh University Gharuan Punjab India

**Keywords:** GLUT‐3, GSK, MAP, PPARγ, diabetic associated neurodegenerations

## Abstract

Flavone derivatives of natural products are often synthesized to enhance their structural specificity, target selectivity, and bioavailability. The current study aimed to examine the neuroprotective efficacy of flavone derivative in diabetic associated neurodegenerations through systematic assessments of in‐silico and in‐vivo. The synthesized flavone (2‐phenyl‐4H‐chromen‐4‐one) was characterized by NMR spectroscopy and FTIR. The in‐vivo assessments were performed by following the serum biochemistry of homeostatic model assessment (HOMA), antioxidant and histopathology of cortex and hippocampus. The in‐silico assessment of molecular docking showed −6.6 Kcal/mol with dipeptidyl peptidase‐4 enzyme (DPP4), −7.8 with acetylcholinesterase (AChE), and −9.5 with butyrylcholinesterase (BuChE). The diabetic neurodegeneration model was induced by the chemical induction method and treated with the test compound at a dose of 40 mg/kg in comparison to sitagliptin. The treatment of the test compound showed significant alterations in the cortex and hippocampus region with mitigated neuronal injuries which endorsed by expressions targeted genes including glucose transporter 3 (GLUT‐3), glycogen synthase kinase 3 beta (GSK‐3β), microtubule associated protein (MAP)‐Tau, and peroxisome proliferator‐activated receptor gamma (PPARγ). Furthermore, the lipid profile and oxidative stress were ameliorated significantly by the course of treatment. In conclusion, the synthesized flavone has significant capability to promote neuroprotective effects in diabetes associated neurodegeneration through mitigating oxidative stress and modulating the expression of the targeted genes, thereby alleviating neuronal injuries.

## Graphical abstract

Neuroprotective efficacy of flavone (2‐phenyl‐4H‐chromen‐4‐one). The study aimed to explore the potential of a synthetic derivative (flavone) of a phytocompound, which is involved in neuroprotection activities. Experimental strategies included the characteristics of compound, in‐silico analysis, and in vivo assessments. The outcomes revealed significantly neuroprotective potential up to molecular interference of the targeted genes along with biochemical and histological interpretations.

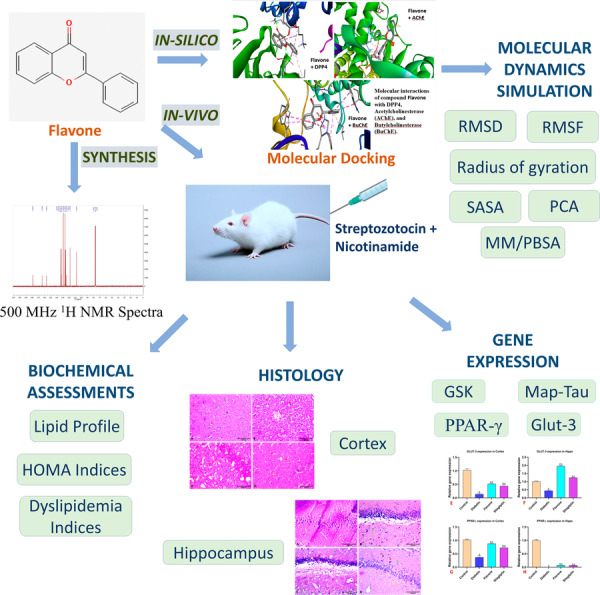

## 1 INTRODUCTION

Flavones (derived from the Latin word flavus, which means yellow) are polyphenolic heterocyclic chemicals found in a wide variety of plants. They are one of the most significant and influential flavonoid subgroups and plant secondary metabolites with a 2‐phenylchromen‐4‐one backbone.^1^ When compared to other flavonoids, flavones are distinguished by their unsaturated carbon ring among C2 and C3 in the flavonoid skeleton, with the absence of a substitutions at the C3 position, and a ketone in the C4 position.^2^, ^3^ Flavones, in addition to their numerous biochemical, physiological, and ecological activities in plants, also have biological functions in animals and are regarded as valuable nutrients. They contain both polar (hydrophilic) and non‐polar (lipophilic) fragments, which allow them to interact with bilayer membranes of cells.^4^ Flavones are a crucial component in numerous nutraceutical, pharmaceutical, medical, and cosmetic applications. They have a broad range of health‐promoting advantages, including anti‐inflammatory, antioxidant, anti‐allergic, anti‐cancer, anti‐microbial, estrogenic, metal‐chelating, and anti‐osteoporotic properties. These kinds of properties govern through the key mechanisms underlying these biological activities is their ability to inhibit several metabolic enzymes, such as xanthine oxidase (XO), cyclooxygenase (COX), lipoxygenase, and phosphoinositide 3‐kinase (PI3K). Supportively, numerous intervention strategies have shown by the uses of flavone through enhancing the occurrence of enzymatic antioxidants enzymes.^5^ Interventions on the flavone‐rich diet demonstrated anti‐diabetic properties, which are coherent with flavone's ability to reduce oxidative stress and regulate inflammation.^1^, ^6^ On the other hand, recent developments in synthetic chemistry have produced several therapeutically significant derivatives of flavone. This work represents the ameliorating potential of the synthesized flavones in diabetes associated neurodegenerations through interfering with the neurodegenerative and metabolic genes.

## 2 MATERIALS AND METHODS

### 2.1 Chemicals and synthesis of flavone

The flavone was synthesized in the Department of Biochemistry, Indian Institute of Technology (IIT), Jodhpur. Streptozotocin, nicotinamide, stains, reagents, and other chemicals were obtained up to the chemical grade of Hi‐media and Loba chemicals, Mumbai, India.

The flavone was synthesized by following the standard protocol through sequential steps.^7^, ^8^

**Figure 1:**
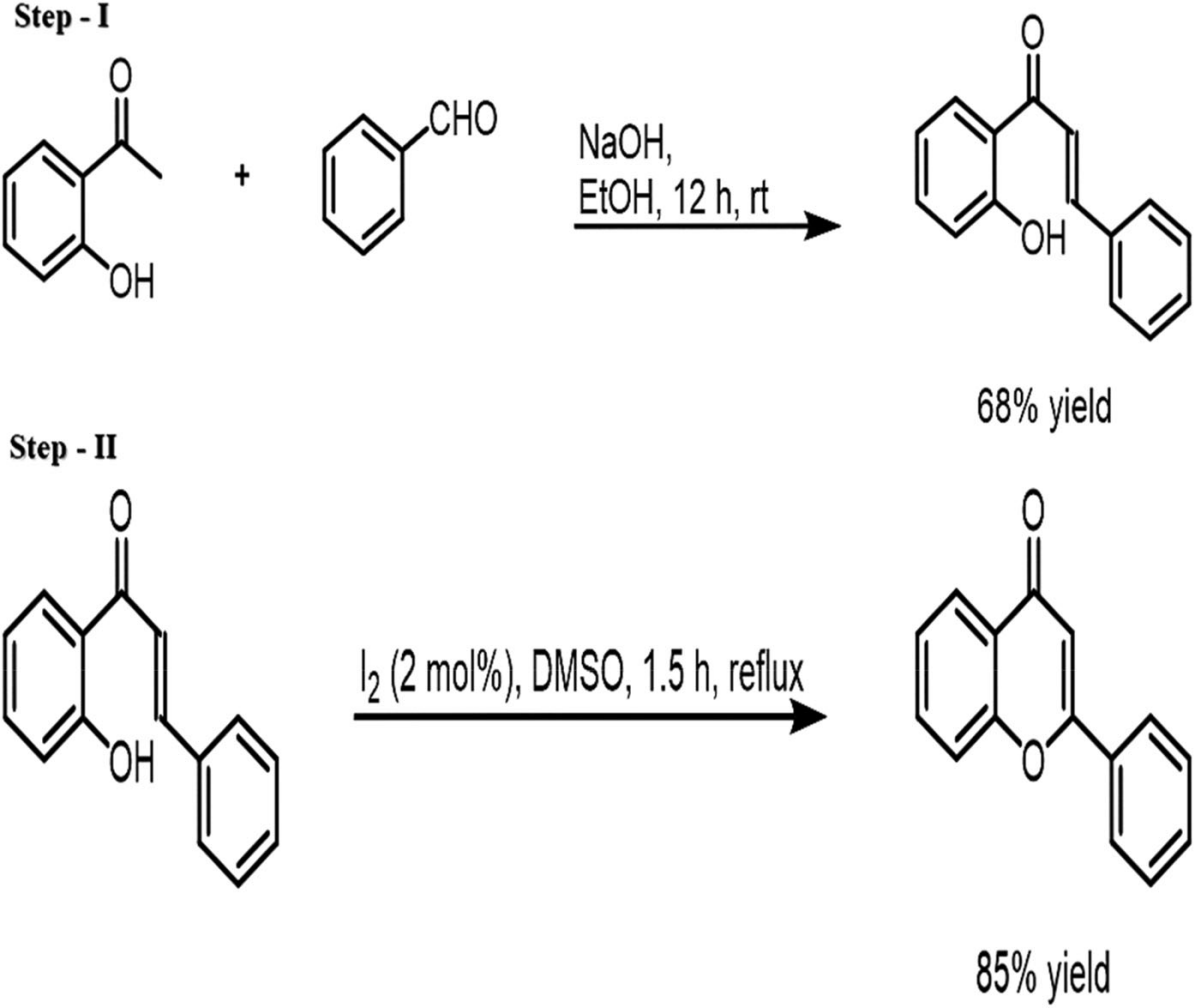
Synthesis process of flavone (2‐phenyl‐4H‐chromen‐4‐one).

In anhydrous ethanol (0.4 M), 1‐(2‐hydroxyphenyl) ethanone (1 equiv.) and benzaldehyde (1.1 equiv.) were stirred for 5 min. Then, NaOH (3 equivalents) was added slowly to the reaction mixture. The reaction mixture was stirred at an ambient temperature and monitored via thin‐layer chromatography. After complete consumption of benzaldehyde, the reaction was quenched with 0.1 N HCl solution until the pH of the reaction medium became acidic in nature. The product was extracted over the addition of ethyl acetate (EtOAc). The organic layer was collected, dried over anhydrous Na_2_SO_4_, and concentrated under reduced pressure. Finally, (*E*)‐1‐(2‐hydroxyphenyl)‐3‐phenylprop‐2‐en‐1‐one was obtained in 68% yield). (*E*)‐1‐(2‐hydroxyphenyl)‐3‐phenylprop‐2‐en‐1‐one (1 equiv.) was taken in dimethyl sulfoxide (DMSO) (0.1 M), with Iodine (0.02 equiv.) in a round‐bottom flask. The reaction mixture was heated at reflux for 1.5 h. Water was then added, and the product was extracted with EtOAc. The organic layer was dried with anhydrous Na_2_SO_4_ and concentrated under reduced pressure. The residue was purified by silica gel column chromatography to obtain pure product (2‐phenyl‐4*H*‐chromen‐4‐one) in 85% yield (Figure 1). More characterization data of compounds can be found in the Supporting Information.

### 2.2 In silico assessments

#### 2.2.1 Molecular docking

The molecular docking was performed to assess the capability of interaction between protein ligand. Ligands were obtained from the PubChem database of the U.S. National Library of Medicine (https://pubchem.ncbi.nlm.nih.gov/), while proteins were acquired from the RCSB Protein Data Bank (PDB) database (https://www.rcsb.org/). Autodock Vina version 1.1.2 was used to perform molecular docking, which involves binding a ligand to a receptor of the dipeptidyl peptidase‐4 enzyme (DPP4) (PDB ID: 5Y7K; crystal structure of human DPP4 in complex with inhibitor 1). Further docked with the neurodegenerative protein acetylcholinesterase (AChE) (PDB ID: 4EY6; crystal structure of recombinant human acetylcholinesterase in complex with (‐)‐galantamine) and the protein butyrylcholinesterase (BuChE) (PDB ID: 4BDS; human butyrylcholinesterase in complex with tacrine). DPP4, AChE, and BuChE were selected as molecular docking targets due to their pharmacological relevance in metabolic and neurodegenerative disorders. DPP4 is involved in glucose regulation and has been implicated in neuroinflammation, while AChE and BuChE play key roles in cholinergic neurotransmission and are closely associated with neurodegenerative disease progression. The Autodock tools provided default grid dimensions for further analysis.^9^, ^10^ The protein–ligand complexes were examined using the binding energy, bond length, number of hydrogen bonds, and interacting residues. The BIOVIA Discovery Studio Visualizer 2021 Client 21.1 was used to analyze the molecular interactions between the protein and ligand.

#### 2.2.2 ADMET predictions

The characteristics of phytocompounds, specifically their physicochemical properties, can greatly impact their effectiveness as pharmaceuticals and industrial chemicals. The effectiveness of phytocompounds depends on their absorption, distribution, metabolism, excretion, and toxicity (ADMET) properties. In order to assess the drug‐likeness of phytocompounds, their pharmacokinetic properties were evaluated using the canonical smile format of the compound from the PubChem database and the SwissADME webserver (http://www.swissadme.ch/) of the Molecular Modeling Group of the Swiss Institute of Bioinformatics (SIB).^11^ To evaluate drug‐likeness, pharmacokinetic properties were obtained using the Lipinski rule of 5, and the results were tabulated to evaluate optimal medication properties.

### 2.3 In vivo study

#### 2.3.1 Experimental animals

Albino healthy male Wistar rats were used with an initial mean body weight of 200 ± 15 g. Animals were kept in polypropylene cages and maintained under standard laboratory conditions (12 h light‐dark cycle maintained at an ambient temperature of 23°C ± 2°C and humidity levels of 45–65%). A standard rat pellet with soaked grams was fed with water *ad libitum*. All the animals were maintained as per the national guidelines from the Committee for the Purpose of Control and Supervision of Experiments on Animals (CCSEA), Government of India and protocols were approved by the Institutional Animal Ethics Committee (IAEC No: JNVU/IAEC/2020/01).

#### 2.3.2 Induction of diabetes associated neurodegenerations

Untreated prolonged type 2 diabetes promotes the development of associated complications, such as diabetic neurodegeneration, by following the different degenerative activities. Type 2 diabetes was induced by following the standard protocol of the chemical method. Accordingly, nicotinamide was dissolved in a normal physiologically saline solution (0.9% NaCl solution), whereas streptozotocin was dissolved in a freshly prepared citrate buffer (0.1 M) with a pH of 4.5. Diabetes was induced in overnight fasted male albino rats by a single intraperitoneal (i.p.) injection of nicotinamide (200 mg/kg) followed by i.p. administration of streptozotocin at the dose of 65 mg/kg with the time interval of 15 min.^12^, ^13^

#### 2.3.3 Collection of blood and estimation of serum glucose

The establishment of type 2 diabetic rats was confirmed through glucose levels of the tail veins by using the glucometers. Rats with more than 200 mg/dL of blood glucose levels were used for further study. Whereas the blood samples of biochemistry were collected from leading aortas by following the standard protocols from euthanized subjects during autopsy.^14^

#### 2.3.4 Experimental groups

Twenty animals were randomly divided into four groups with six animals in each group for the experimental duration of 28 days. Flavone was dissolved in olive oil, while sitagliptin was dissolved in water, and both were administered orally by the intra‐gastric route. Animals were divided into the following groups:

1. Vehicle control‐ Normoglycemic control (Group‐I)
2. Diabetic control‐ Hyperglycemic control (Group‐II)
3. Diabetic + Flavone (40 mg/kg) treatment (Group‐III)
4. Diabetic + Standard drug (Sitagliptin) treatment (Group‐IV)

#### 2.3.5 Dose and duration

Sitagliptin (10 mg/kg/day) and flavone (40 mg/kg/day) were both administered daily for 28 days during the experiment. The dose was calculated as per data of mean lethal dose (LD50) and previous studies.^15^ After 28 days of treatment, overnight fasted rats were autopsied. The rats were euthanized by chloroform followed by rapid cervical dislocation and decapitation. The blood was collected by using the capillary from the retro‐orbital sinus, centrifuged (3000 rpm/15 min) at room temperature, and the serum was collected and stored at −4°C for further biochemistry estimation. The brain (hippocampus and frontal cortex) tissues were separated out, washed in ice‐cold saline to remove the blood, patted dry, and placed in a 10% neutral buffered formalin solution.

### 2.4 Serum biochemical analysis

#### 2.4.1 Estimation of serum glucose, total protein, lipid profile, and dyslipidemia indices

The serum glucose,^16^ total protein (TP),^17^ total cholesterol (TC),^18^ triglyceride (TG),^19^ low‐density lipoprotein (LDL) cholesterol, very low‐density lipoprotein (VLDL) cholesterol, and high‐density lipoprotein (HDL),^20^ were quantified spectrophotometrically using enzymatic kits (ERBA Diagnostic Pvt. Ltd.) by enzymatic photo colorimetric methods. The data were reported in mg/dl. Dyslipidemia indices, including Atherogenic Index of Plasma (AIP), Castelli Risk Index‐I (CRI‐I), Castelli Risk Index‐II (CRI‐II), and Atherogenic Coefficient (AC), were assessed using a lipid profile.^21^

#### 2.4.2 Estimation of antioxidants

The oxidative stress was calculated from serum samples by assessments of lipid peroxidation (LPO),^22^ reduced glutathione (GSH),^23^ and Ferric reducing antioxidant potential assay (FRAP),^24^ which was done utilizing total protein (TP) data by following the standard methods.

#### 2.4.3 Homeostatic model assessment (HOMA) analysis

The HOMA indices, that is, HOMA‐IR, HOMA‐β, and HOMA‐S were calculated by using the levels of fasting glucose and insulin as per the following formulas.^25^

$$\text{HOMA}-\text{IR}=\frac{\text{Fasting Insulin}\;\left(U/L\right)\times \text{Fasting Glucose}(\text{mmol}/L)}{22.5},$$

$$\text{HOMA}-\beta \;=\frac{20\;\text{x fasting Insulin}\;(U/L)}{\text{fasting Glucose}\;\left(\text{mmol}\;/L\right)}-3.5,$$

$$\text{Insulin sensitivity}\;(\text{IS})=\frac{1}{\left[\left(\text{Insulin}\;(U/L)\;\text{xLog}\;(\text{glucose}\;(\text{mmol}/L)\right)\right]}.$$

### 2.5 Histological examination

The tissues were further processed by using standard procedures and embedded in paraffin wax for further histological examination.^26^, ^27^ The brain (hippocampus and frontal cortex) sections were cut coronally using a rotary microtome at a thickness of 5 µm. These sections were further dehydrated in ethanol, stained with hematoxylin and eosin dye (H&E), and mounted in a neutral deparaffinated xylene medium to assess histopathological changes, and observations were made with a clinical microscope. The sections were captured using the microscope's built‐in camera.

### 2.6 Molecular biology of the gene expression

#### 2.6.1 RNA isolation and cDNA synthesis

The frontal cortex and hippocampus were extracted from each group using the Trizol method.^28^, ^29^ The extracted RNA was quantified spectrophotometrically using a NanoDrop One^C^ (Thermo Scientific). The samples used were chosen based on their A260/280 ratio, which ranged from 1.6 to 2.0. RNA was preserved at −80°C for future use. cDNA synthesis (reverse transcription) was performed using the commercially available kit qScript® cDNA Synthesis (Quanta BioSciences) following the manufacturer's instructions.

#### 2.6.2 Gene expression quantification

Quantitative real‐time polymerase chain reaction (qPCR) analysis was performed using PerfeCTa® SYBR® Green FastMix® (Quanta BioSciences). The dilution method and a temperature gradient reaction from 50°C to 65°C were used to determine primer efficiencies.^30^, ^31^ The total reaction volume was 10 µL, consisting of 5 µL of Perfecta SYBR Green FastMix (2X), 0.5 µL each of gene‐specific forward and reverse primers, 1.5 µL of nuclease‐free water, and 2.5 µL of cDNA. The CFX96 Real‐Time System manufactured by Bio‐Rad was employed for qPCR analysis, utilizing the following cycle parameters: an initial temperature of 95°C for 30 s, followed by 39 cycles of 95°C for 5 s, 60°C for 15 s, and 68°C for 15 s. The utilization of melting curves was employed in the analysis of qPCR products to ascertain the presence of non‐specific products or primer dimer formation (Table 1). The experiment involved the analysis of each sample in triplicate. The calculation of relative gene expression for individual genes was performed using the equation 2^−ΔΔCT. This methodology ensures accuracy and consistency in the results obtained, making it a reliable approach. The results were adjusted by using GAPDH as a reference gene.

**Table 1:** Primer sequences used for gene expression analysis.

S No.	Gene	Primer sequences (5′→3′)	No of bases
1.	GSK	F‐ GAGAGCTCCAGATCATGAGAAAG	23
		R‐ CTTAACCTGGTGCTGGACTATG	22
2.	MAP Tau	F‐ AAGAAGCAGGCATCGGAGAC	20
		R‐ CCTTGGCTTTCTTCTCGTCA	20
3.	GLUT‐3	F‐ AACAGAAAGGAGGAAGACCA	20
		R‐ CGCAGCCGAGGGGAAGAACA	20
4.	PPAR‐γ	F‐ TGGGGATGTCTCACAATGCC	20
		R‐ AGACTCTGGGTTCAGCTGGT	20
5.	GAPDH	F‐ AGGTCGGTGTGAACGGATTTG	21
		R‐ TGTAGACCATGTAGTTGAGGTCA	23

### 2.7 Statistical analysis

The data of the biochemical evaluations were expressed as a mean ± standard error of the mean (SEM). Statistical analysis was performed using GraphPad Prism software (version 8.0.2). Two‐way analysis of variance was used for statistical analysis, and the statistical variances were calculated before the post hoc mean separation test. *p* < 0.05 was regarded as statistically significant.

## 3 RESULTS

### 3.1 In silico evaluation

#### 3.1.1 Molecular docking

Molecular docking was performed to analyze the interactions of flavone with DPP4, AChE, and BuChE in comparison to the conventional drug sitagliptin. The Autodock Vina results revealed a binding energy of −6.6 Kcal/mol with DPP4, −7.8 with AChE, and −9.5 with BuChE (Table 2). Further processing of the proteins and ligands using Discovery Studio Visualizer was conducted to determine the number of hydrogen bonds, bond length, and interacting residues. The interacting residues of flavone with DPP4 included Ala291, Ile295, Lys696, Gln697, and Arg317, Glu693. Similarly, with AChE, the interacting residues were Val429, Val408, and Val330. With BuChE, the interacting residues were Trp82, Leu286, Trp231, Gly116, Phe329, His438, and Ser198. These findings provide valuable insights into the molecular interactions of flavone with DPP4, AChE, and BuChE.

**Table 2:** Molecular interaction studies of the flavone against the target enzyme.

Protein	Dipeptidyl peptidase‐4 enzyme		Acetylcholinesterase		Butylcholinesterase	
Ligand	Flavone	Sitagliptin	Flavone	Sitagliptin	Flavone	Sitagliptin
Binding energy (Kcal/mol)	−6.6	−6.9	−7.8	−9.6	−9.5	−10.3
No. of H‐bonds	8	6	8	15	11	13
Bond length (Å)	(4.09,4.85), 5.38,(3.95,4.39), 2.61,2.89,4.35	3.67,3.54, (3.48, 3.84,3.42),3.47	4.51,(4.34,2.74, 2.95,2.45), 3.76,5.13,5.44	2.20,3.29,2.66, 1.82,5.14,3.28, 3.03,3.62,(2.22, 2.35,2.67,4.65, 3.68,3.35,3.67)	(5.33,4.90),4.93, (5.06,4.97),5.39, 5.08,(3.73,4.56, 5.24),2.04	4.97,3.50,4.90, 3.61,3.38,(4.15, 3.63),3.50,5.43, (2.98,3.96,4.15), 4.84
Interacting residues	Ala291, Ile295, Lys696, Gln697, Arg317, Glu693	Gln761, Gln731, Asp729, His757	Val429, Val408, Val330	Gln527, Ala526, Tyr510, Glu431, Leu386, Gly523, Leu524, Val429, Arg525	Trp82, Leu286, Trp231, Gly116, Phe329, His438, Ser198	Gly116, Gly117, Phe329, His438, Gly439, Trp82, Pro285, Phe398, Trp231, Leu286

#### 3.1.2 ADMET predictions

The flavone was subjected to a comparison of its characteristics with those of the standard drug, sitagliptin (Table 3). The findings indicate that the flavone exhibits an ideal drug profile and does not violate Lipinski's rule of Five, which signifies that it possesses drug‐like properties. Furthermore, the compound's total polar surface area value was found to be 30.21, a parameter that indicates cellular plasma membrane permeability.

**Table 3:** Physiochemical ADMET analysis of the flavone against the Lipinski rule of five and blood‐brain barrier filter by the SwissADME web tool.

Compound	Flavone	Sitagliptin
MW	222.24	407.31
ilogP	2.55	2.35
ClogP	3.18	2.51
MlogP	2.27	2.52
HBA	2	10
HBD	0	1
nHB	2	11
TPSA	30.21	77.04
Nviolations	0	0
Drug likeness	Yes	Yes
Filter L/B	L/B	L/B

Abbreviations: ClogP, consensus log P; ilogP, partition coefficient; Filter L/B, Lipinski rule of five and B, blood‐brain barrier; HBA, hydrogen bond acceptor; HBD, hydrogen bond donor; MlogP, Moriguchi octanol‐water partition coefficient; MW, molecular weight; nHB, number of hydrogen bonds; TPSA, total polar surface area; nviolations, number of violations.

### 3.2 Flavone treatment improved insulin resistance, β‐cell function, and glycemic control

**Figure 2:**
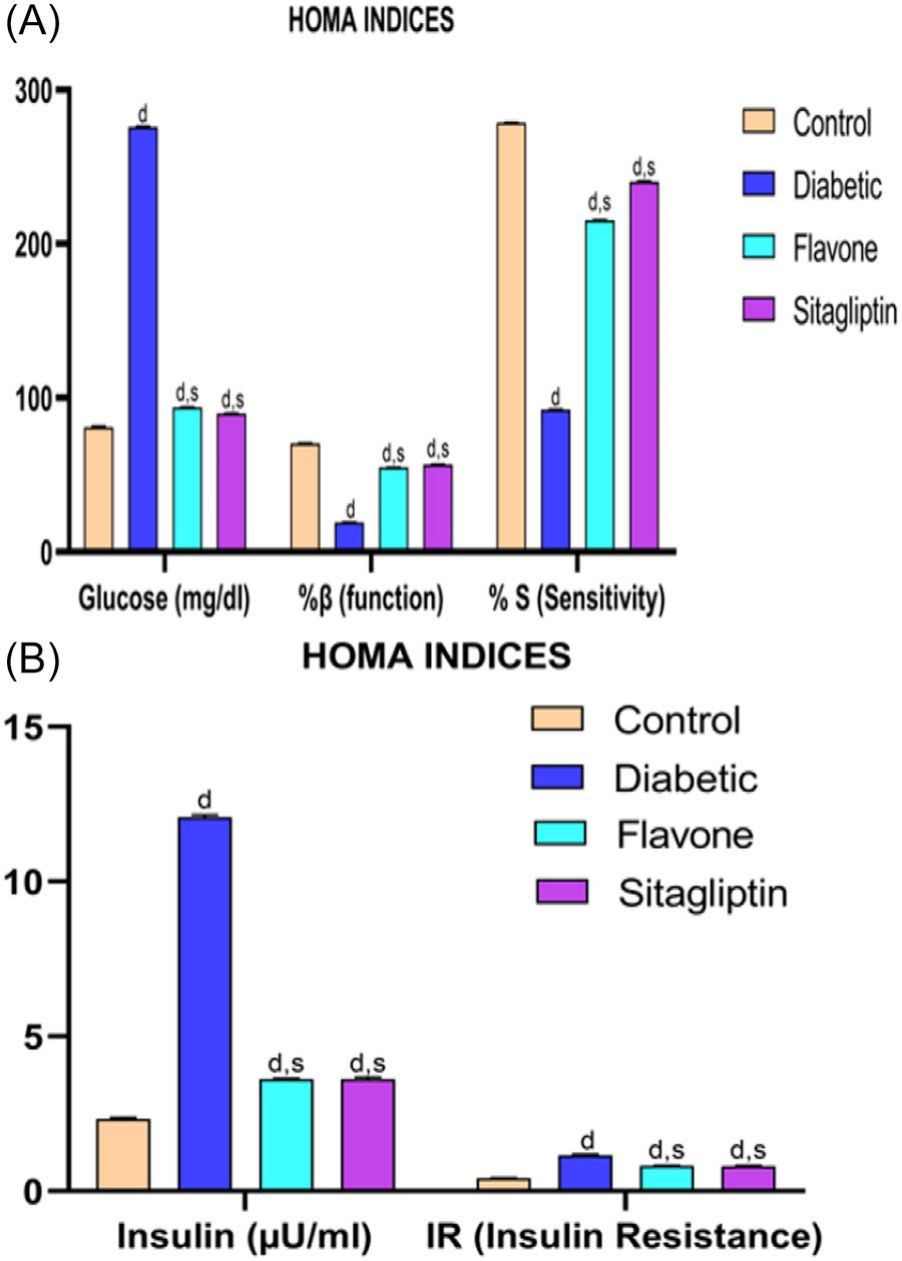
Variations in HOMA indices across different groups. (A) Fasting glucose, HOMA‐β (%β‐cell function), and HOMA‐%S (insulin sensitivity). (B) Fasting insulin and HOMA‐IR (insulin resistance index). Data are presented as means ± SEM (*n* = 6); statistical analysis was done using two‐way ANOVA. d represents a significant difference with *p* ≤ 0.0001 compared with the control group; s represents a significant difference with *p* ≤ 0.0001 compared with the diabetic group.

HOMA assessments revealed that insulin resistance was much higher in the diabetic control group, whereas treatment with flavone and sitagliptin reduced insulin resistance significantly (*p* ≤ 0.0001). Concurrently, β‐cell function and insulin sensitivity rose dramatically in the flavone and sitagliptin administration groups. The compound flavone significantly (*p* ≤ 0.0001) improved glucose and insulin levels in type 2 diabetic rats (Figure 2A and 2B).

### 3.3 Flavone treatment improved lipid profile and dyslipidemia indices

**Figure 3:**
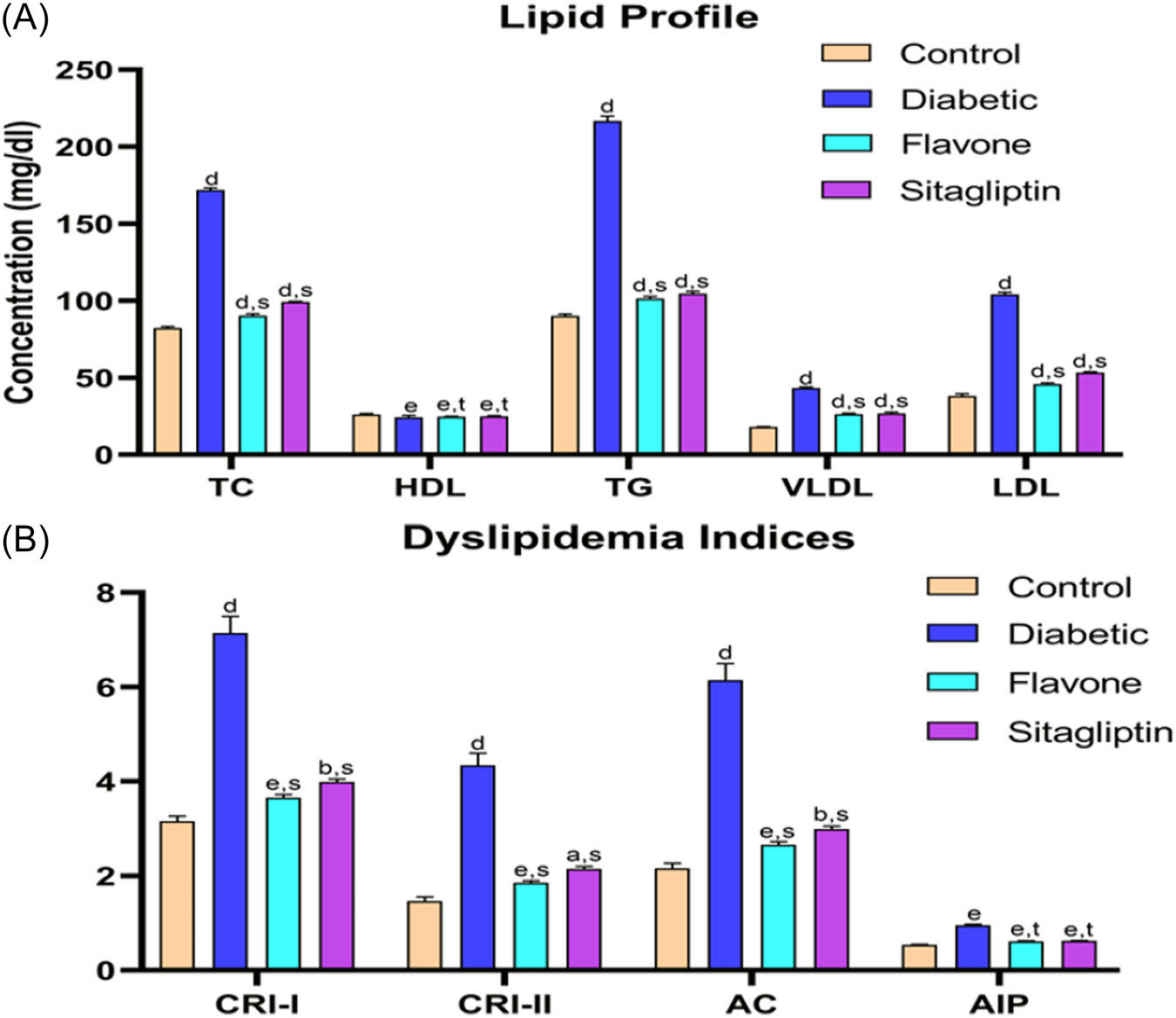
Variations in lipid profile and dyslipidemia indices across different groups. (A). TC, HDL, TG, VLDL, and LDL levels. (B). CRI‐I, CRI‐II, AC, and AIP index. Data are presented as mean ± SEM (*n* = 6); statistical analysis was done using two‐way ANOVA. a, b, and d represent *p* ≤ 0.1, ≤0.01, ≤0.0001 compared with the control group; s represents a significant difference with *p* ≤ 0.0001 compared with the diabetic group; e and t indicate non‐significant differences compared with the control and diabetic groups, respectively. AC, Atherogenic Coefficient; AIP, Atherogenic Index of Plasma; CRI‐I, Castelli Risk Index‐I; CRI‐II, Castelli Risk Index‐II; HDL, high‐density lipoprotein; LDL, low‐density lipoprotein; TG, triglyceride; TC, total cholesterol; VLDL, very low‐density lipoprotein.

The diabetes control group had significantly (*p* ≤ 0.0001) increased TC, LDL cholesterol, VLDL cholesterol, and TG levels compared with the vehicle control groups. However, flavone significantly decreased TC, LDL cholesterol, VLDL cholesterol, and TG in diabetic rats (Figure 3A). Furthermore, the indices of dyslipidemia, such as the AC, CRI‐I, and CRI‐II, were reduced compared with the diabetes control group (Figure 3B).

### 3.4 Flavone treatment ameliorated oxidative stress

**Figure 4:**
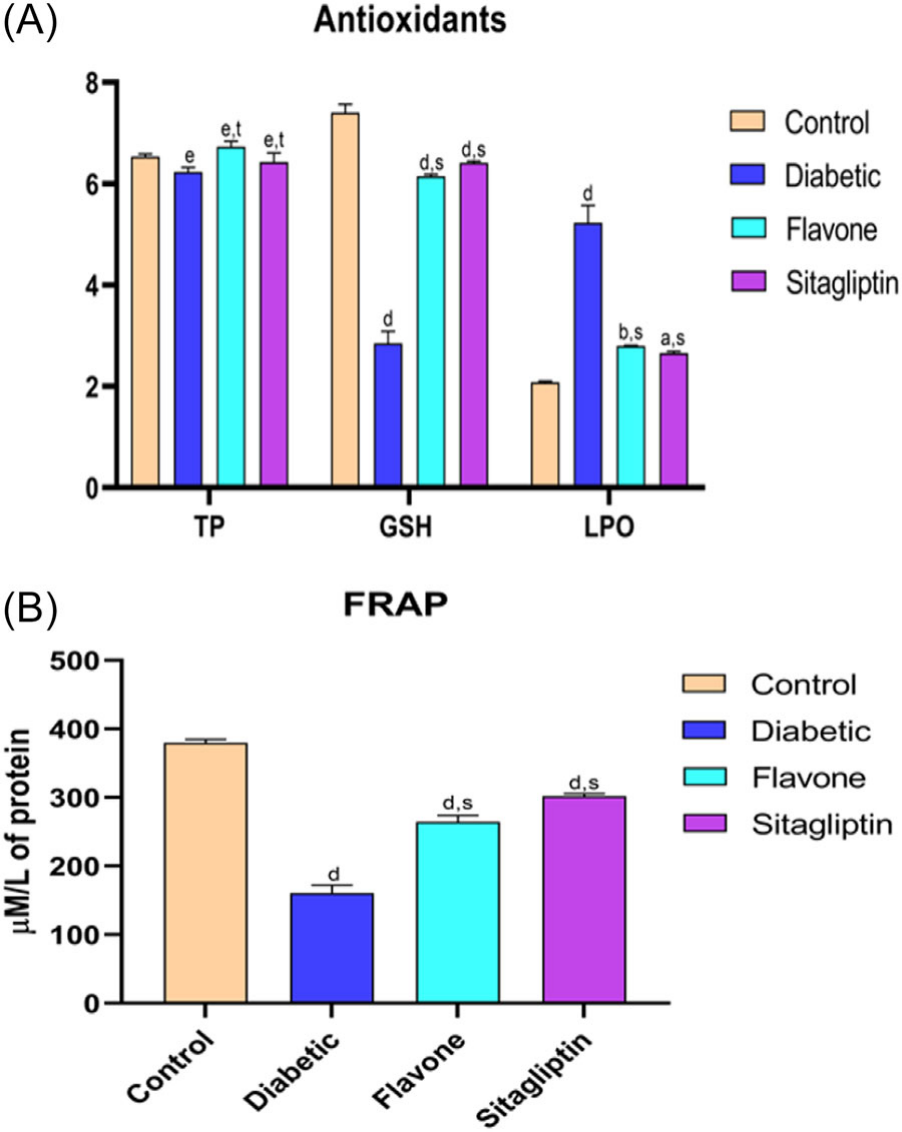
Variations in antioxidant parameters across different groups. (A) Antioxidants level. (B) FRAP. Data are presented as mean ± SEM (*n* = 6); statistical analysis was done using two‐way ANOVA. a, b, and d represent *p* ≤ 0.1, ≤0.01, ≤0.0001 compared with the control group respectively; s represents a significant difference with *p* ≤ 0.0001 compared with the diabetic group; e and t indicate non‐significant differences compared with the control and diabetic groups, respectively. FRAP, Ferric reducing antioxidant potential assay; LPO, Lipid peroxidation; TP, Total protein; GSH, reduced glutathione.

In comparison to the animals in the vehicle control group, the diabetes control group's levels of LPO were much greater, whereas GSH levels and FRAP were significantly lower (*p* ≤ 0.0001). In comparison to the diabetic control group, the flavone‐treated diabetic rats had significantly (*p* ≤ 0.0001) higher levels of GSH and lower levels of LPO, and improved FRAP assay (Figure 4). These findings indicated that the diabetic state produced a marked pro‐oxidative shift in systemic redox balance. However, the flavone‐treated diabetic rats showed significantly higher GSH and lower LPO and FRAP assay values compared with the diabetic control group, indicating that flavone administration reduced lipid oxidative damage while replenishing intracellular thiol‐based antioxidant reserves. The observed increase in GSH implies either enhanced GSH synthesis or decreased GSH consumption due to lower ROS burden, whereas the reduced LPO provides direct evidence of diminished membrane lipid oxidation following treatment. Together, these changes are compatible with an overall antioxidant and membrane‐protective effect of the tested flavone in the diabetic model.

### 3.5 Flavone treatment attenuated diabetes‐induced neuronal injury

**Figure 5:**
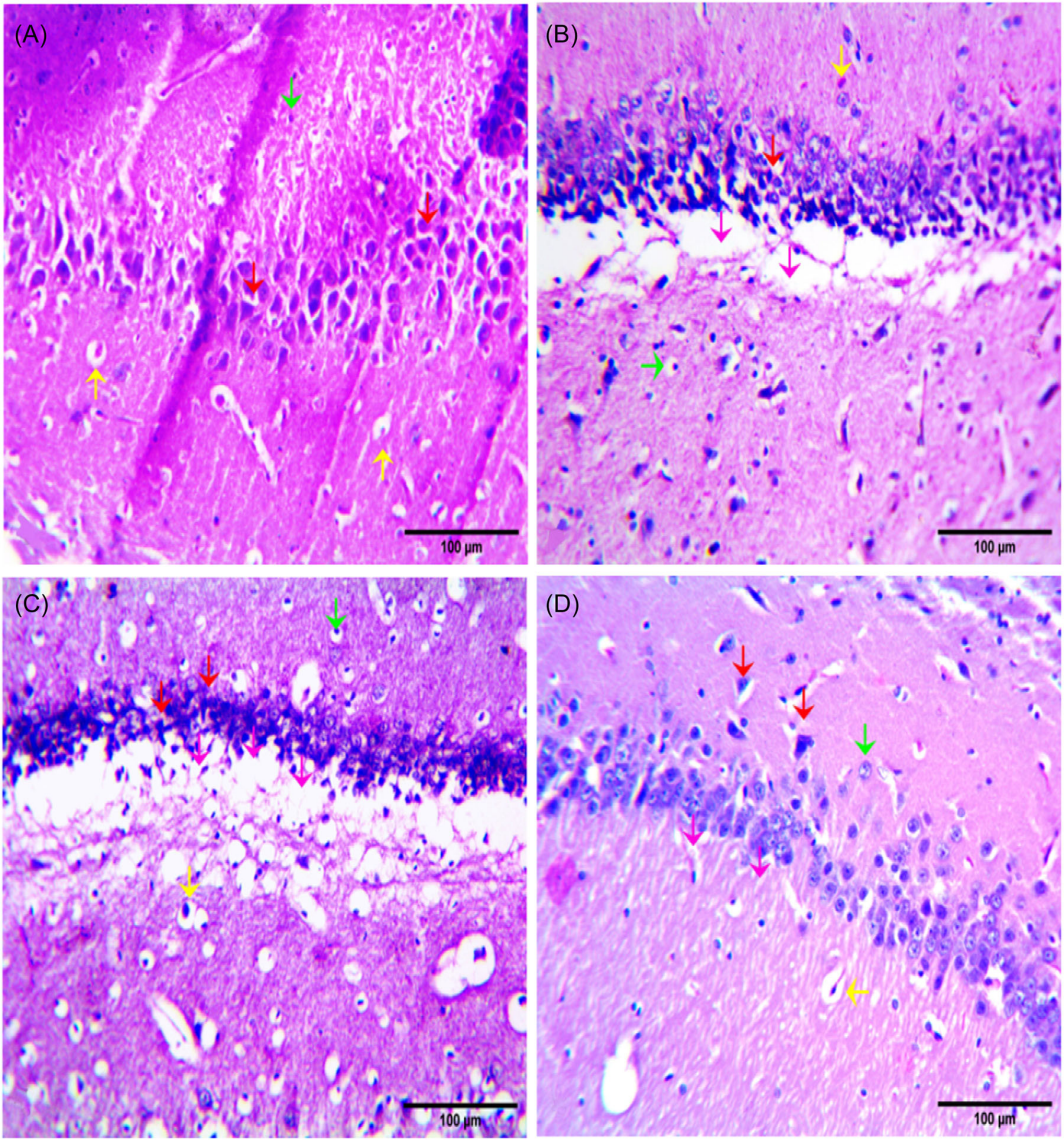
Histological architecture of rat hippocampus stained with haematoxylin and eosin (H&E). (A) Vehicle control. (B) Diabetic control. (C) Flavone‐treated group. (D) Sitagliptin‐treated group. The red arrows show pyramidal cells, the green arrows indicate neuronal cells, the yellow arrows show a large focal area of gliosis and the pink arrows indicate degeneration and neuronal necrosis (Magnification = 100× and scale bar = 100 µm for images).

Histopathological analysis of the hippocampus in the CA3 region in the diabetic group reveals enlarged pericellular spaces and chromatolysis, indicating neuronal cell injury or neurodegeneration. The control group has normal neuronal cells with distinct cell layers, the sitagliptin group has partial restoration of neuronal cells and layers, and the flavone compound‐treated group shows restoration of neuronal cells with distinct characteristic cell layers (Figure 5).

**Figure 6:**
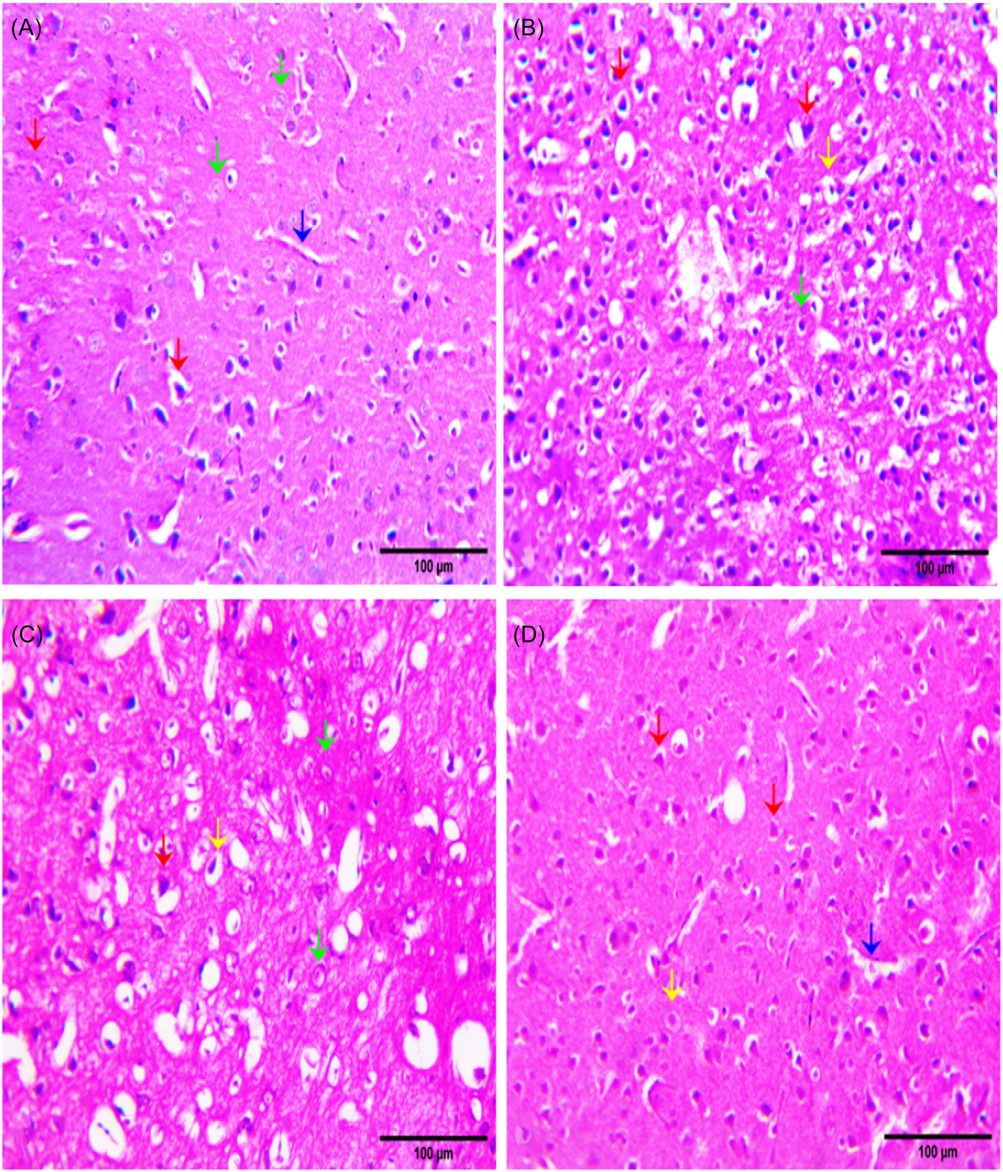
Histological architecture of rat cortex stained with haematoxylin and eosin (H&E). (A) Vehicle control. (B) Diabetic control. (C) Flavone‐treated group. (D) Sitagliptin‐treated group. The red arrows show pyramidal cells; the green arrows indicate neuronal cells; the yellow arrows show a large focal area of gliosis; and the blue arrows indicate degeneration and neuronal necrosis (Magnification = 100× and scale bar = 100 µm for images).

Histopathological analysis of the frontal cortex in the diabetic group showed vacuolization and reduced dark cells. Apoptotic cells can be recognized by their pyknotic nuclei, constricted cytoplasm, and circular halos. The control group has smaller glia cells and blood capillaries scattered in between the neurons, and the pyramidal cells displayed open‐face nuclei and basophilic cytoplasm. The standard sitagliptin shows scattered glia cells and open‐face nuclei, while the flavone compound‐treated group showed pyramidal cells with nearly normal morphology, basophilic vesicular nuclei, and prominent nucleoli appeared (Figure 6). However, pyramidal cells that were undergoing apoptosis were still present.

### 3.6 Flavone treatment regulated the expression of neurodegenerative and metabolic genes

**Figure 7:**
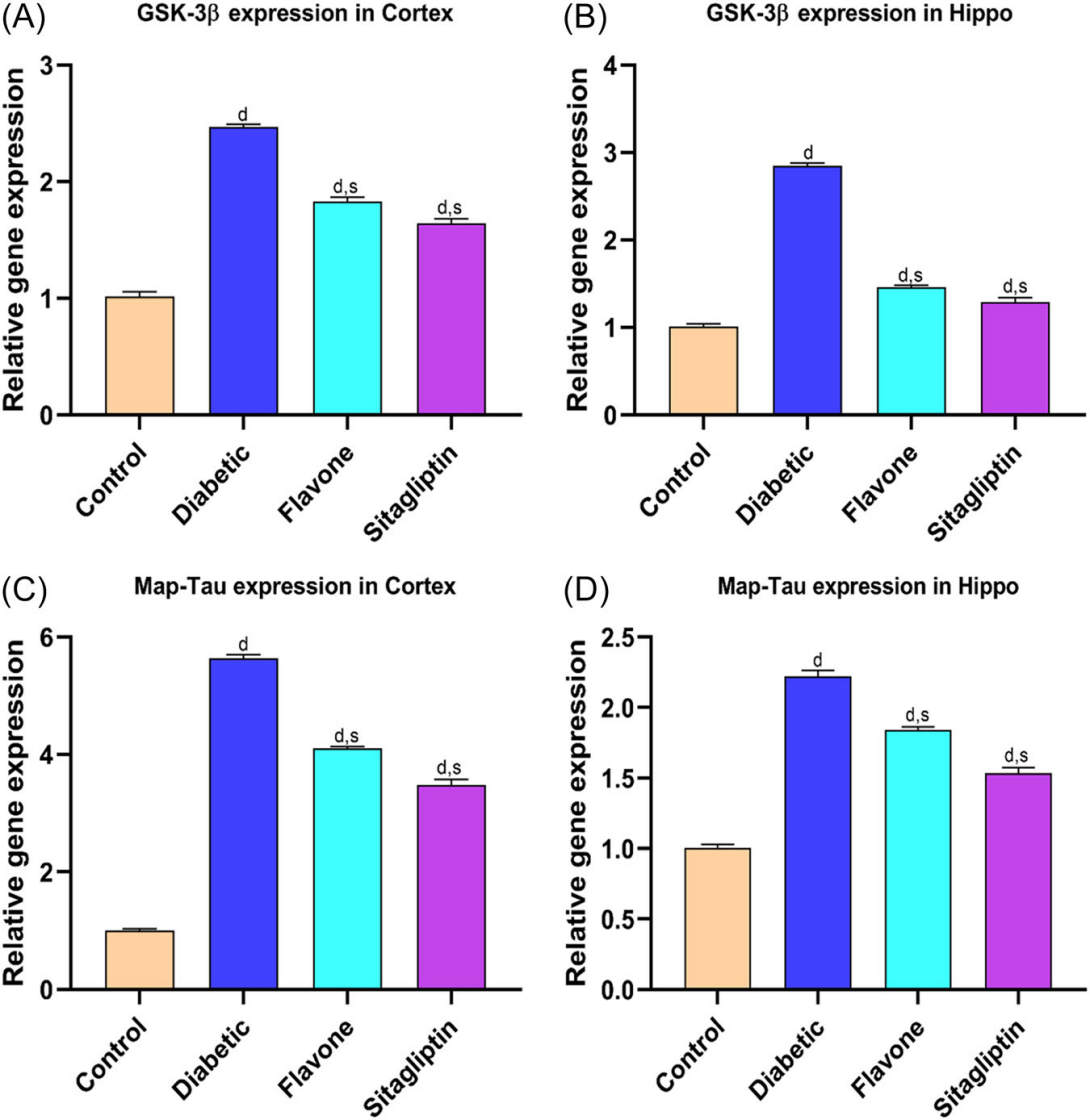
Comparison of mean changes in the gene expression levels of GSK‐3β and MAP‐Tau in the cortex and hippocampus of different rat groups. (A, B) GSK‐3β expression levels in the cortex and hippocampus, respectively. (C, D) MAP‐Tau expression levels in the cortex and hippocampus, respectively. Data are presented as mean ± SEM (*n* = 6); statistical analysis was done using two‐way ANOVA. d represents a significant difference with *p* ≤ 0.0001 compared with the control group; s represents a significant difference with *p* ≤ 0.0001 compared with the diabetic group.

**Figure 8:**
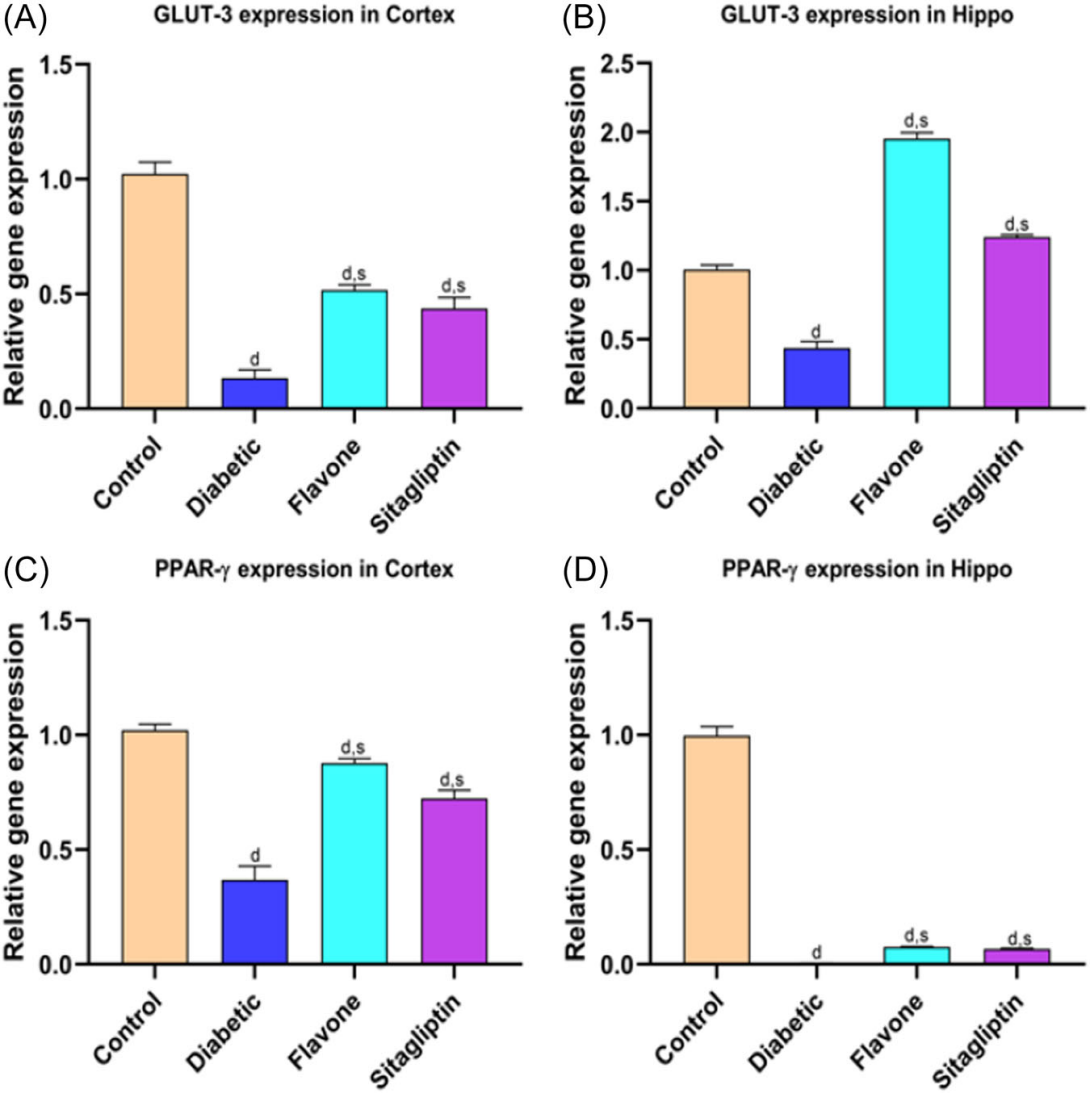
Comparison of mean changes in the gene expression levels of GLUT‐3 and proliferator‐activated receptor gamma (PPAR‐γ) in the cortex and hippocampus of different rat groups. (A, B) GLUT‐3 expression levels in the cortex and hippocampus, respectively. (C, D) PPAR‐γ expression levels in the cortex and hippocampus, respectively. Data are presented as mean ± SEM (*n* = 6); statistical analysis was done using two‐way ANOVA. d represents a significant difference with *p* ≤ 0.0001 compared with the control group; s represents a significant difference with *p* ≤ 0.0001 compared with the diabetic group.

qPCR analysis was utilized to assess the expression of certain neurodegenerative and metabolic genes, such as glycogen synthase kinase 3 beta (GSK‐3β), microtubule‐associated protein (MAP)‐Tau, glucose transporter 3 (GLUT‐3), and peroxisome proliferator‐activated receptor gamma (PPAR‐γ), in the frontal cortex and hippocampus tissues of rats that were subjected to four different treatments. The diabetic groups showed a significant upregulation of GSK‐3β and MAP‐Tau expression, accompanied by a downregulation of GLUT‐3 and PPAR‐γ in both the frontal cortex and hippocampus compared with the control group. However, treatment with flavone significantly reversed these diabetes‐induced alterations. In the frontal cortex, flavone administration was associated with a clear downregulation of GSK‐3β and MAP‐Tau, accompanied by an upregulation of GLUT‐3 and PPAR‐γ when compared with the diabetic control group. Similar regulatory trends were observed in the hippocampus, where flavone treatment reduced the expression of GSK‐3β and MAP‐Tau while enhancing GLUT‐3 and PPAR‐γ expression (Figures 7 and 8).

## 4 DISCUSSION

Insulin resistance in neural tissues causes reduced uptake of glucose, which sequentially promotes apoptosis followed by the related consequences resulted in neurodegenerations.^32^, ^33^ Diabetic neuropathy is the leading complication of diabetes, which is observed as memory loss and other ill sensations. The neurodegenerations and apoptotic activities are associated with the oxidative stress along with abnormal HOMA indices. The similar kinds of observations revealed in type 2 diabetic animal model as reported by earlier researchers.^34^, ^35^ In the diabetic brain, oxidative stress induced by hyperglycemia is crucial for neuronal damage. In the present investigation, hyperglycemia was induced using streptozotocin‐nicotinamide, which destroyed insulin activity and disrupted the control of plasma glucose. Further, the current study reveals that the treatment of flavone in the animal model of diabetic neuropathy led to significant improvements in insulin levels, oxidative stress, and the histopathology of the brain (hippocampus and frontal cortex), in addition to a significant decrease in blood glucose and HOMA indices with the regulation of neurodegenerative and metabolic genes.

Molecular docking is an incredibly effective technique utilized for identifying new drugs that target receptors with known structures. It is a vital aspect of structure‐based drug design and computer‐aided drug discovery, which involves screening small molecules that are positioned and evaluated in a protein's binding site. The ultimate outcome of this process is the creation of innovative ligands for receptors.^36^, ^37^ The focus of this study involved the docking of flavone into the active site of the DPP4, AChE, and BuChE proteins that play crucial roles for neurodegeneration, with the aim of gaining a better understanding of its structural basis for the activity of neuroprotection. This research is of particular significance as it provides valuable insight into the potential applications of flavone, as well as its mechanisms of action. By examining the interactions between flavone and the DPP4, AChE, and BuChE protein, we can enhance our understanding of this compound's potential as a therapeutic agent. The complex achieved its established conformation through H‐bond interactions. The presence of a larger number of hydrogen bonds indicates a stronger binding affinity between the ligand and the receptor.^38^ ADMET predictions further confirmed its drug‐like properties with a good cellular plasma membrane permeability. Overall, these findings suggest that flavone has potential as a therapeutic agent for these proteins.

The pancreatic β‐cell necrosis caused by streptozotocin, which promotes ATP dephosphorylation and causes the subsequent formation of free radicals (superoxide, hydrogen peroxide, and hydroxyl radicals), may be the cause of the rise in serum glucose and concurrent drop in serum insulin levels.^39^, ^40^ The anti‐diabetic properties of flavone may be mediated by the reduction of oxidative stress. Treatment with flavone in diabetic rats halted the progression of diabetes by either reducing intestinal glucose absorption or by enhancing the glycolytic and glycogenic pathways, with a concomitant decrease in the glycogenolysis and gluconeogenesis pathways.^41^ Flavone revealed beneficial effects by lowering the plasma glucose levels, implying that this may have an impact on the metabolic pathways for glucose. In this study, we found that administering flavone orally at 40 mg/kg body weight for 28 days to diabetic animal model caused abnormal blood glucose and serum insulin levels to normalize. It unequivocally and effectively restored the lipid profile, dyslipidemia indices, and antioxidant levels to almost normal levels. HOMA analysis demonstrating the restoration of β‐cell function in flavone‐treated streptozotocin‐diabetic rats supports the possibility that flavone boosted insulin response through either antioxidant action or the regeneration of β‐cells. As a result of the functional restoration of insulin‐producing cells, this increases the insulinotropic impact of flavone.

Due to its fast metabolic rate, high lipid content, and the relative absence of an antioxidant enzyme, the brain is particularly sensitive to oxidative damage.^42^, ^43^ Histopathology of the hippocampus and frontal cortex reversed the results of diabetic rats when administered with flavone and showed that it could arrest the progression of neuronal damage in diabetic rats by regulating GSK‐3β, MAP‐Tau, GLUT‐3, and PPAR‐γ. The PPAR‐γ has been established as a crucial regulator of adipocyte differentiation, fatty acid storage, and glucose metabolism. It is targeted by several antidiabetic drugs due to its role in enhancing gene expression encoding proteins that are involved in glucose and lipid metabolism.^27^, ^44^ Notably, PPAR‐γ plays important roles in neuroprotection via the antioxidant response, anti‐inflammatory, lipid metabolism, and immune regulation processes. GSK‐3β is a vital protein that plays a crucial role in regulating metabolic and signaling processes in the human body. It has been identified as a potent inhibitor of glycogen synthase, an enzyme that facilitates the conversion of glucose to glycogen. Upon binding with insulin, GSK‐3β receptors activate the PI3K/Akt pathway. However, overexpression of GSK‐3β has been shown to impair glucose tolerance and reduce glycogen synthesis, ultimately affecting insulin signaling. It is therefore imperative to identify the regulatory mechanisms that govern GSK‐3β activity to maintain the optimal balance of glucose metabolism and insulin signaling in the body.^45^, ^46^ GLUT3, which was initially discovered in a fetal muscle cell line, has been identified as the primary glucose transporter in the neural elements of the brain. MAP tau is a crucial protein factor for microtubule assembly, with the highest expression in human brain neurons.^47^ Subsequently, significant alterations were observed in the expression of PPAR‐γ, GSK‐3β, GLUT3, and MAP tau in the cortex and hippocampus of brain tissues as reported by the earlier studies. Therefore, flavone can be considered a promising supplementary medication for preventing neurodegeneration.

## 5 CONCLUSION

Flavone has the potential to improve the diabetes associated neurodegeneration by targeting the inhibition of key enzymes, which resulted in the regulation of neurodegenerative and metabolic genes. The supplementation of flavone also ameliorates the oxidative stress and mitigates diabetes‐induced neuronal injuries in the cortex and hippocampus. Therefore, further investigations with varying doses and treatment durations in higher animal models are warranted to validate its efficacy and explore its potential as a potent neuroprotective agent.

## AUTHOR CONTRIBUTIONS

Karishma Sen, Anita Sakarwal, and Sunil Dutt Shukla conducted the in vivo experiments. Heera Ram supervised the study and was responsible for the study design. Suman K. Saha and Nirmal K. Rana performed the compound synthesis. Dharmveer Yadav, Mukesh Kumar Yadav, and Balachandar Vellingiri carried out the molecular biology experiments. Vikas Kumar edited and proofread the manuscript. All authors read and approved the final manuscript.

## CONFLICT OF INTEREST STATEMENT

The authors declare no conflicts of interest.

## ETHICS STATEMENT

Protocols of in vivo experimentations were approved by the Institutional Animal Ethical Committee (IAEC No: JNVU/IAEC/2020/01), Department of Zoology, JNVU, Jodhpur (Rajasthan), India, as per the considerations of CCSEA (The Committee for Control and Supervision of Experiments on Animals), India.

## Supporting information

Flavone_compound_supporting.

## Data Availability

The data supporting the findings of this study are available from the corresponding author upon reasonable request.
