# Supplementary material for: Synthesized flavone attenuates diabetes‐induced neurodegeneration through regulation of oxidative stress and metabolic‐neurodegenerative molecular pathways
Source: Ibrain. 2026 Apr 18;12(2):259–72. doi: 10.1002/ibra.70019 (PMC13310239; doi:10.1002/ibra.70019)
Supplement: Supplementary file 1 — Flavone_compound_supporting. [file IBRA-12-259-s001.docx]

1. **Characterization data of compounds:**
2. **(E)-1-(2-hydroxyphenyl)-3-phenylprop-2-en-1-one**: yellow solid; yield = 68%; purified by

flash chromatography with hexane/ethyl acetate (19:1) as eluents and silica gel (100-200 mesh) as stationary solid phase; ^1^H NMR (500 MHz, CDCl_3_) δ 12.81 (s, 1H), 7.96 – 7.91 (m, 2H), 7.69 – 7.65 (m, 3H), 7.53 – 7.49 (m, 1H), 7.47 – 7.42 (m, 3H), 7.07 – 7.01 (m, 1H), 6.98 – 6.93 (m, 1H); ^13^C NMR (125 MHz, CDCl_3_) δ 193.90, 163.74, 145.63, 136.56, 134.75, 131.08, 129.80, 129.19, 128.81, 120.27, 120.16, 119.01, 118.79; IR (Neat) ν 3034, 2961, 2931, 1641, 1586, 1574, 1487, 1443, 1343, 1306, 1207, 1157, 1021, 863 cm^-1^; HRMS (ES+) calc. for C_15_H_13_O_2_+ [M+H]^+^: 225.0910, found: 225.0916; Melting point: 77-79 °C.

1. **2-phenyl-4H-chromen-4-one**: white solid; yield = 85% ; purified by flash chromatography with hexane/ethyl acetate (9:1) as eluents and silica gel (100-200 mesh) as stationary solid phase; ^1^H NMR (500 MHz, CDCl_3_) δ 8.24 (d, *J* = 7.9 Hz, 1H), 7.93 (d, *J* = 7.1 Hz, 2H), 7.70 (t, *J* = 7.7 Hz, 1H), 7.55 (dd, *J* = 21.1, 7.4 Hz, 4H), 7.42 (t, *J* = 7.5 Hz, 1H), 6.83 (s, 1H); ^13^C NMR (125 MHz, CDCl_3_) δ 178.61, 163.56, 156.41, 133.91, 131.92, 131.74, 129.18, 126.43, 125.85, 125.37, 124.10, 118.22, 107.73; IR (Neat) ν 3018, 1636, 1607, 1571, 1465, 1379, 1218, 1217, 908, 851 cm^-1^; HRMS (ES+) calc. for C_15_H_11_O_2_+ [M+H]^+^: 223.0754, found: 223.0766; Melting point: 197-199 °C.
2. **^1^H and ^13^C NMR Spectra:**


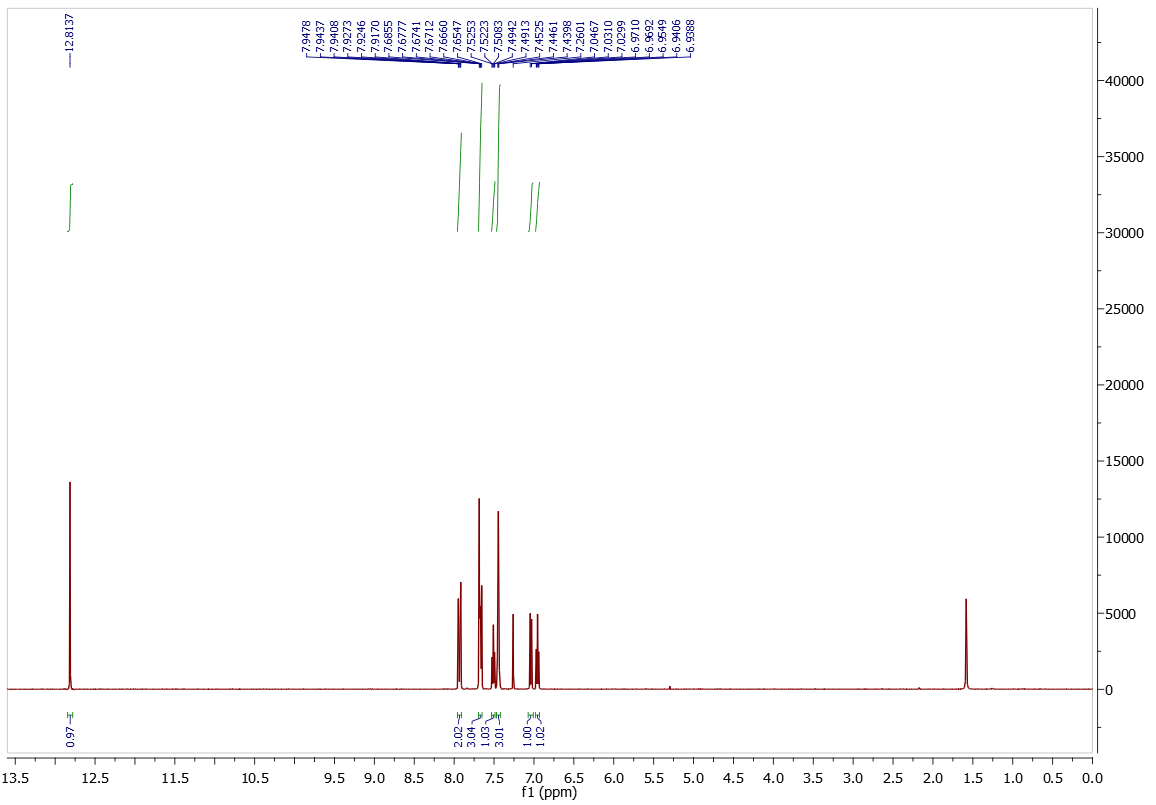


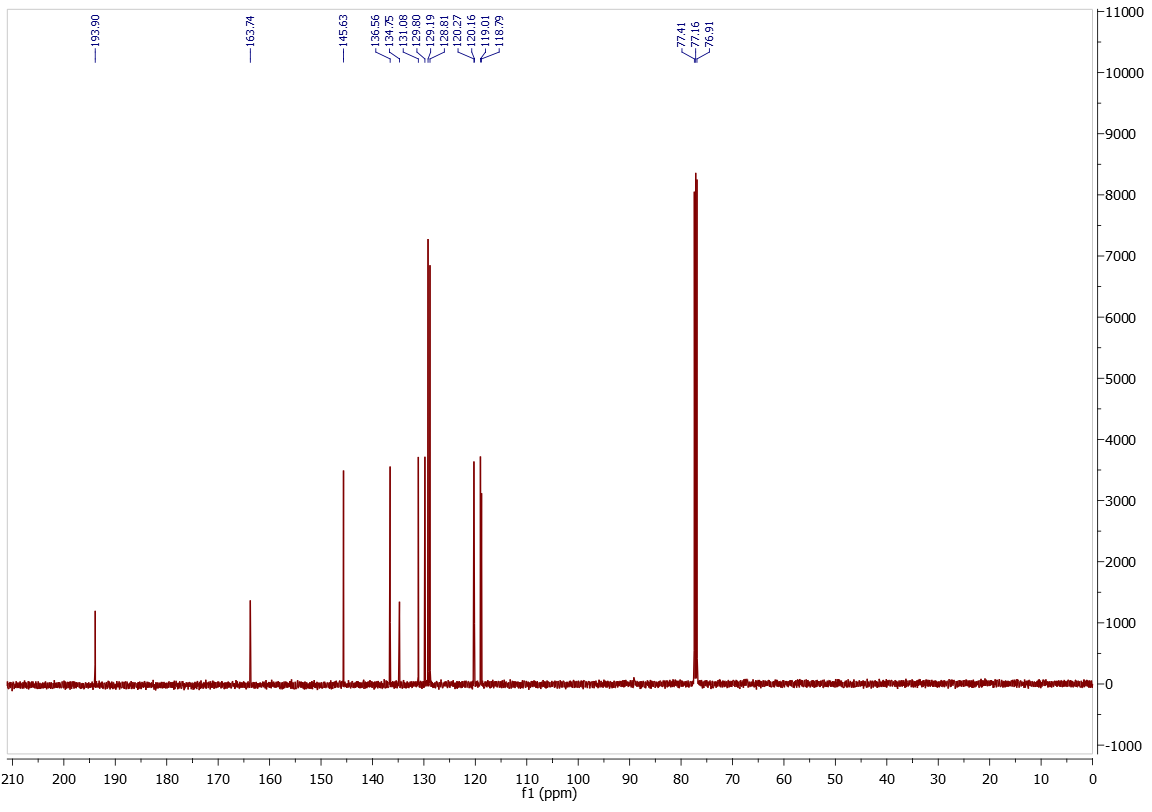


**500 MHz ^1^H NMR and 125 MHz ^13^C NMR Spectra**


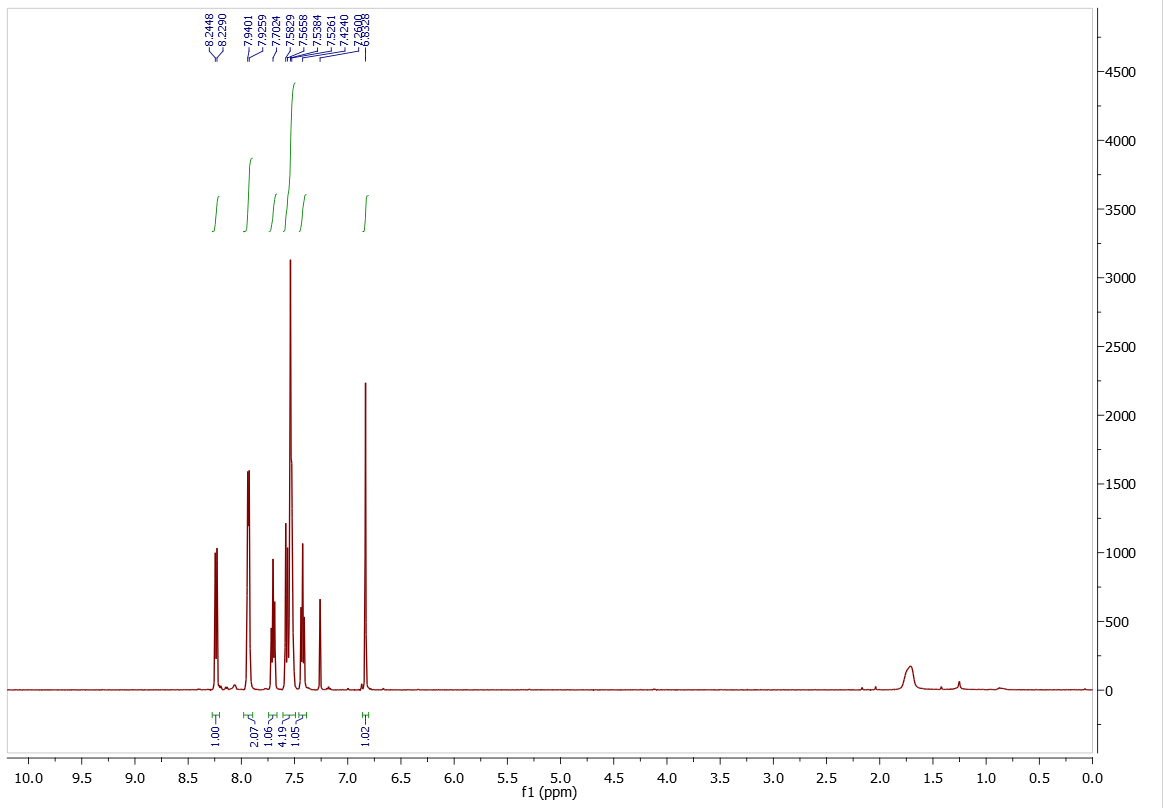

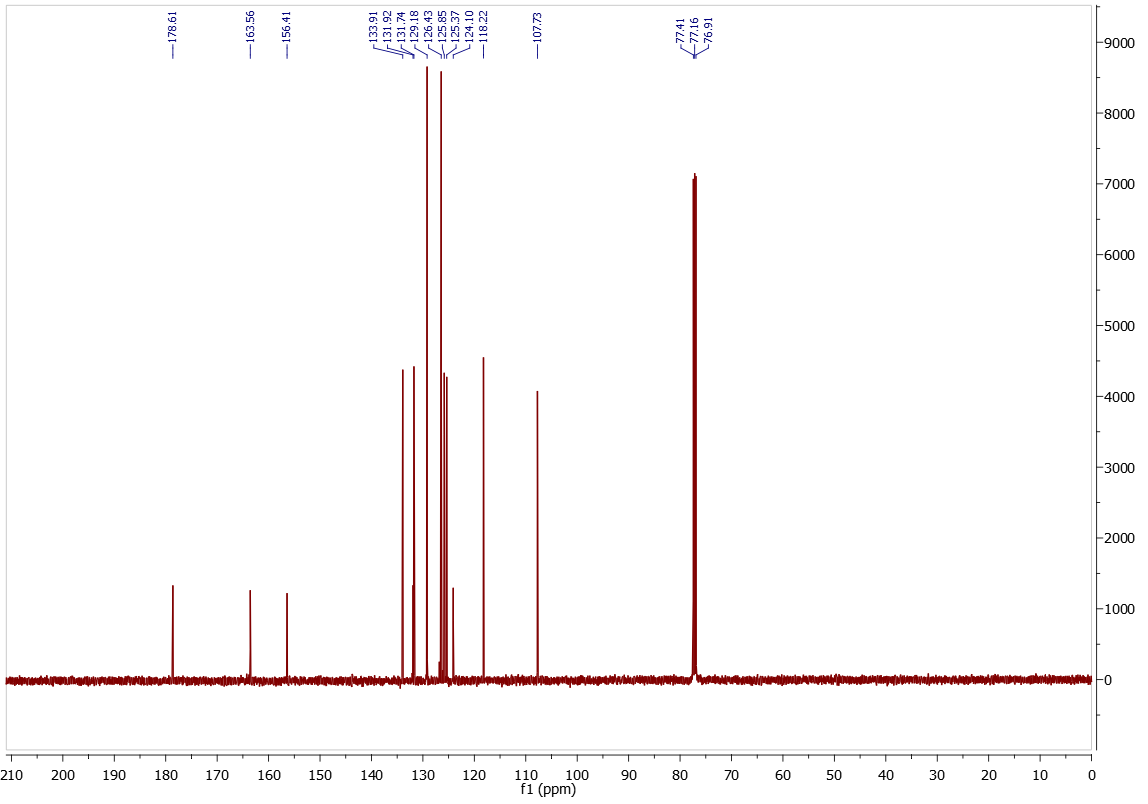


**500 MHz ^1^H NMR and 125 MHz ^13^C NMR Spectra**

1. **HRMS Spectra:**

**
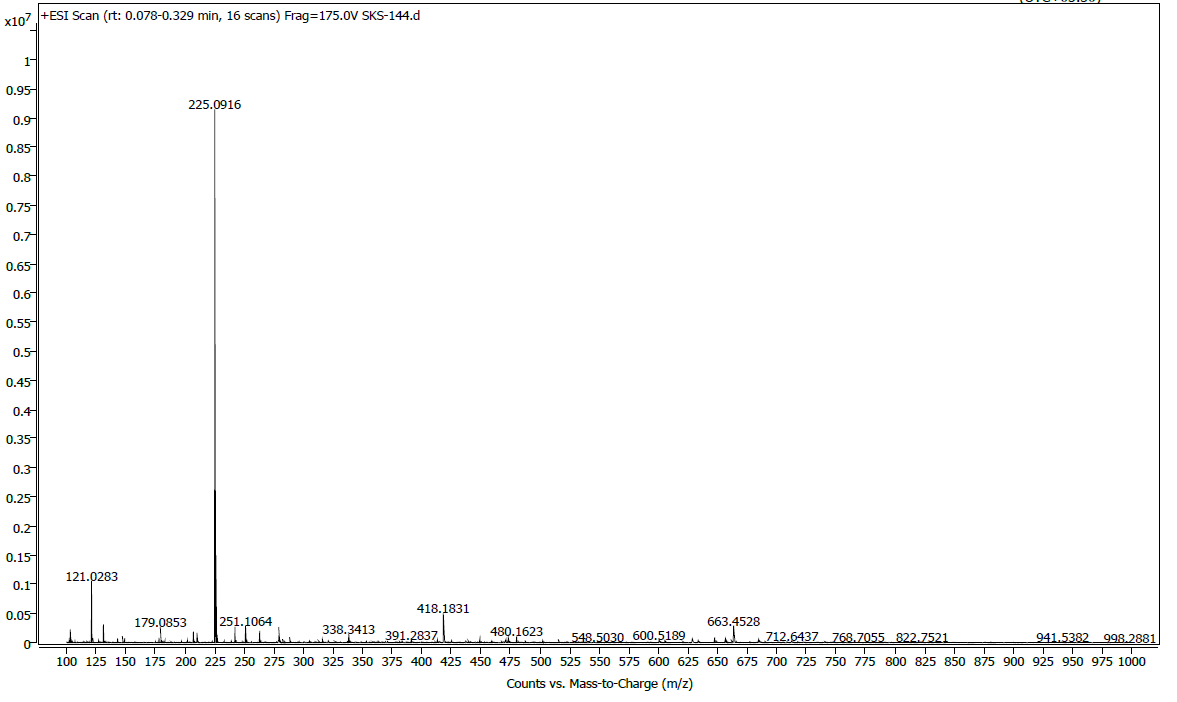
**

**
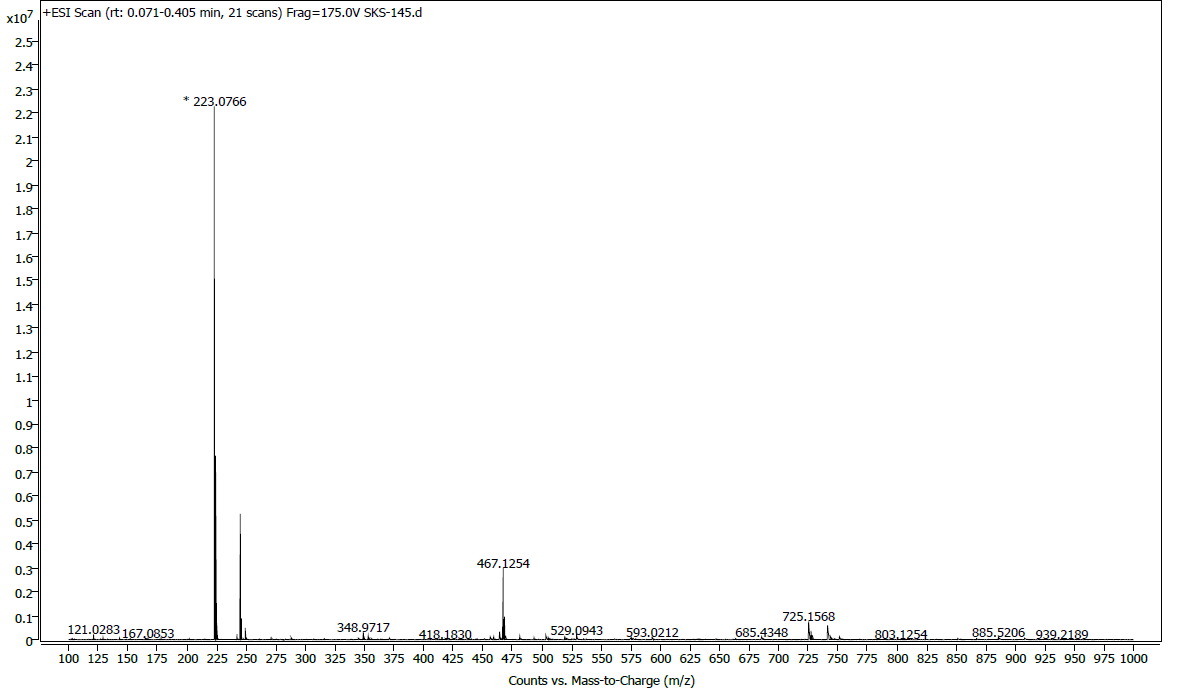
**

1. **IR Spectra:**

**
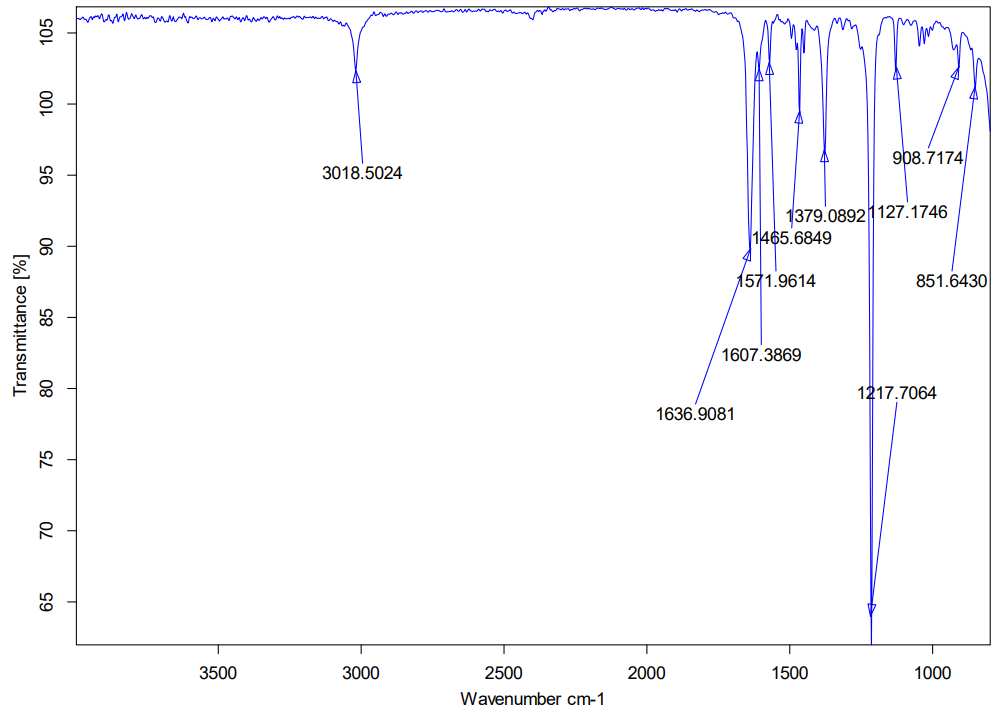
**

**References**

1. Cabrera, M.; Simoens, M.; Falchi, G.; Lavaggi, M. L.; Piro, O. E.; Castellano, E. E.; Vidal, A.; Azqueta, A.; Monge, A.; Cerain, A. L. D.; Sagrera, G.; Seoane, G.; Cerecetto, H.; Gonzalez, M. Synthetic chalcones, flavanones, and flavones as antitumoral agents: Biological evaluation and structure–activity relationships *Bioorg. Med. Chem.* **2007**, *15*, 3356.

2. Shan, G.; Yang, X.; Ma, L.; Rao, Y. Pd-Catalyzed C-H Oxygenation with TFA/TFAA: Expedient Access to Oxygen-Containing Heterocycles and Late-Stage Drug Modification. *Angew. Chem., Int. Ed.* **2012**, *51*, 13070−13074.
